# Les transcrits bcr-abl et leurs corrélations avec l’hémogramme au cours de la leucémie myéloïde chronique (LMC) au Togo

**DOI:** 10.11604/pamj.2018.30.221.9821

**Published:** 2018-07-20

**Authors:** Essohana Padaro, Hèzouwè Magnang, Yao Layibo, Koffi Mawussi, Irénée Messanh Kuéviakoé, Kossi Agbétiafa, Ahoéfa Vovor

**Affiliations:** 1 Service d’Hématologie, CHU Campus, Université de Lomé, Togo; 2 Service d’Hématologie, CHU Tokoin, Université de Lomé, Togo; 3 Service d’Hématologie, CHU Kara, Université de Kara, Togo

**Keywords:** Leucémie myéloïde chronique, bcr-abl, Lomé, Togo, Chronic myeloid leukemia, bcr-abl, Lomé (Togo)

## Abstract

Décrire les différents types de transcrits de fusion moléculaire bcr-abl afin d'évaluer leur fréquence et étudier leurs influences sur les paramètres de l'hémogramme au diagnostic. Il s'agit d'une étude prospective de 34 patients porteurs d'une leucémie myéloïde chronique au Togo. La recherche des transcrits de fusion a été réalisée au laboratoire d'hématologie biologique de l'hôpital Henri Mondor à Créteil (France). L'âge moyen des patients était de 42,32±14,87 ans avec des extrêmes de 9 et 65ans. On notait une prédominance masculine avec un sex- ratio de 1,61 (21 hommes et 13 femmes). L'étude moléculaire révélait deux types de transcrits b3-a2 et b2-a2. Dix-neuf patients (55,88%) avaient le transcrit b3-a2, 13 (38,24%) le transcrit b2-a2 (32,10 %) et deux patients possédaient les deux types de transcrits b3-a2 et b2-a2 (5,88%). Au diagnostic, le taux d'hémoglobine moyen, le nombre moyen de globules blancs, le chiffre moyen des plaquettes chez les patients porteurs du transcrit b3-a2 étaient respectivement de 99,2g/l ; 207,63G/l et 451,28G/l. Pour les patients porteurs du transcrit b2-a2 les valeurs sont 104,6g/l, 114,32G/l et 486,11G/l. Les patients ayant les deux types de transcrit de fusion avaient respectivement 67g/l, 867G/l et 780G/l. Les paramètres de l'hémogramme sont significativement plus perturbés chez les patients porteurs des deux types de transcrits b3-a2 et b2-a2.

## Abstract (en)

This study aims to describe the different bcr-abl gene transcript variants in order to determine their frequency and to study their influence on CBC diagnostic test. We conducted a cross-sectional study of 34 patients with chronic myeloid leukemia in Togo. The search for fusion transcripts was performed in the laboratory of biological haematology at the Henri Mondor Hospital, Créteil (France). The average age of patients was 42,32±14,87 years ranging between 9 and 65 years. Most patients were male, with a sex- ratio of 1.61 (21 men and 13 women). Molecular examination showed b3-a2 transcript and b2-a2 transcript. Nineteen patients (55.88%) expressed b3-a2 transcript, 13 patients (38.24%) b2-a2 transcript (32.10%) and two patients expressed both b3-a2 and b2-a2 transcripts (5.88%). At diagnosis, mean hemoglobin level, the average number of white blood cells and the average number of platelets in patients expressing b3-a2 transcript were 99,2g/L; 207,63g/l and 451,28g/l respectively. In patients expressing b2-a2 transcript values were 104,6g/l, 114,32g/l and 486,11g/l. In patients with both transcripts, values were 67g/L, 867g/l and 780g/l respectively. CBC parameters are more significantly altered in patients with both transcripts b3-a2 and b2-a2.

## Introduction

La leucémie myéloïde chronique (LMC) est un syndrome myéloprolifératif caractérisé par une prolifération prédominante de la lignée granuleuse, la présence d'une anomalie chromosomique, le chromosome Philadelphie (Ph1). Le chromosome Philadelphie correspond à une translocation réciproque et équilibrée (sans perte de matériel génétique) entre les bras longs des chromosomes 9 et 22. Il est noté t(9,22) (q 34, q11) [1]. Ce remaniement chromosomique aboutit à la fusion de deux gènes bcr et c-abl et à la synthèse d'ARN messagers et des protéines bcr-abl hybrides. Le diagnostic de cette hémopathie repose principalement sur l'hémogramme, le myélogramme et sur des méthodes cytogénétiques et moléculaires [2]. Deux types de transcrits sont possibles : b2-a2 dans 25 à 30 % des cas et b3-a2 dans 55 à 60 % des cas. La mise en évidence du chromosome Ph est indispensable dans la majorité des cas et revêt une importance particulière dans l'évaluation de la maladie résiduelle chez certains patients [2]. L'introduction de l'imatinib mesylate (Glivec*, Novartis) utilisé pour la première fois en clinique en juin 1998 a révolutionné le traitement des patients atteints de LMC Ph+. En ciblant la tyrosine kinase ABL constitutionnellement activée chez ces patients, le traitement par l'imatinib permet d'atteindre 89 % de survie globale entre 0 et 5ans. Soixante-neuf pourcent des patients traités par imatinib atteignent une réponse cytogénétique en 1 an avec disparition du chromosome Ph1 et une réponse hématologique à 3 mois [1]. Au Togo [3], la LMC représente 24,52% des hémopathies malignes avec une incidence annuelle de 3 cas. Notre travail a pour objectif de décrire les différents types de transcrits observés chez les patients LMC suivis au CHU-Campus de Lomé et leurs influences sur les principaux paramètres de l'hémogramme lors du diagnostic de la maladie.

## Méthodes

Il s'agissait d'une étude prospective portant sur trente-quatre patients suivis pendant une période de 10 ans pour une LMC au CHU-Campus de Lomé. Il était réalisé systématiquement chez tous les patients : un examen physique complet, une exploration biologique avec hémogramme, un myélogramme, un bilan biochimique, une exploration morphologique (échographie abdominale, radiographie pulmonaire de face) ; une exploration fonctionnelle (électrocardiogramme). Après un consentement éclairé daté et signé, un prélèvement de 2,5ml de sang veineux prélevé sur tube PaxGène^®^ était fait et adressé au service d'hématologie biologie du CHU Henri Mondor de Créteil (France). Le réarrangement chromosomique bcr-abl a été mis en évidence chez tous les patients en RT-PCR. Il s'agit d'une étude quantitative du transcrit BCR- ABL par PCR quantitative. Le résultat est exprimé en recherche positive ou négative, précise le point de cassure, la sensibilité testée de l'analyse, la qualité de l'ARN et le pourcentage M BCR- ABL/ABL.

## Résultats

Sur un total de 1821 patients (en majorité des drépanocytaires) suivis par le service d'hématologie clinique du CHU-Campus de Lomé, 34 patients avaient une leucémie myéloïde chronique, soit 01,86 % de tous les patients. La prévalence annuelle de la LMC est donc d'environ 3,4 cas par an en consultation d’hématologie. L'âge moyen des patients était de 42,32 ± 14,87 ans avec des extrêmes de 9 et 65 ans. Il existait une prédominance masculine (21 hommes et 13 femmes) avec une sex-ratio de 1,61. La moyenne des paramètres de l'hémogramme au diagnostic montraient un taux moyen en hémoglobine de 100,6±2,59 G/L (extrêmes : 57 et 147G/L), le nombre moyen des globules blancs était de 195± 19,09G/L (extrêmes : 64 et 971G/l) avec un nombre moyen des polynucléaires neutrophiles à 88,55G/L, celui des plaquettes était de 451,66±20,41G/L (extrêmes : 51 et 1116G/L). L'étude moléculaire au diagnostic révélait la présence des deux types de transcrits b3-a2 et b2-a2. Dix-neuf patients (55,88%) avaient le transcrit b3-a2, 13 (38,24%) le transcrit b2-a2 (32,10 %) et deux patients possédaient les deux types de transcrits b3-a2 et b2-a2 (05,88%). La différence des fréquences entre b3-a2 et b2-a2 est significative (p ≤0, 01). Il existait une prédominance de transcrit b3-a2 au diagnostic. Pour tous les patients la sensibilité testée de l'analyse était de 10-4 et la qualité de l'ARN était correcte. La moyenne du % M BCR- ABL/ABL était de 37.3660 (extrêmes : 0.3554-94.6094). La numération blanche (NB) en fonction du type de transcrit, montre une différence significative (Tableau 1). Il existe également une différence significative entre les taux d'hémoglobine (Hb) et le type de transcrit (Tableau 2). La différence entre taux d'hémoglobine au diagnostic et type de transcrits est significative avec Chi2 = 30,28 ; ddl = 15 ; p = 0,111. L'étude de la corrélation entre le type de transcrit et les paramètres de l'hémogramme montre que pour les patients ayant le transcrit b3- a2, le taux d'hémoglobine moyen, le nombre moyen de leucocytes, le nombre moyen de plaquettes étaient respectivement de 99,2g/l±1,76 (extrêmes 65 et 127), 207,631G/l ±125,650 (extrêmes 8,3 et 524G/l) et 451,287G/L±292,420 (extrêmes 108 et 1116G/L). Pour les patients ayant le transcrit b2-a2, ces valeurs sont respectivement de 104,6 g/l±16,7 (extrêmes 80 et 146g/l) ; 114,321G/L ±80,559 (extrêmes 6,4 et 236,1G/L) et 486,111G/L±191,405 (extrêmes 175 et 779G/L). Les deux patients présentant les deux types de transcrits avaient en moyenne un taux d'hémoglobine de 67 g/l, un nombre de globules blancs de 867 G/L et le nombre de plaquettes était 780G/L. Il existait une relation statiquement significative entre le type de transcrit et la perturbation des paramètres de l'hémogramme. En effet, les anomalies étaient plus profondes chez les patients ayant les deux types de transcrits suivies des patients ayant le b3-a2 et enfin de ceux ayant le b2-a2.

**Tableau 1:** Répartition des patients en fonction de la NB et du type de transcrit

	<100 (G/L)	100-200 (G/L)	200-300 (G /L)	>300(G/L)	Total
b 3-a2	2	6	6	2	16
b 2-a2	4	4	1	0	9
b2-a2 et b3a2	0	0	0	1	1
Total	8	10	7	3	28

La différence est très significative. Chi2 = 36 ,42 ; ddl = 12 ; p = 0,03

**Tableau 2:** Répartition des patients en fonction du taux d’Hbet du type de transcrit

	< 80 (g/l)	80 -100 (g/l)	100 –120 (g/dl)	> 120 (g/dl)	Total (%)
b3-a2	2	5	6	3	16 (57,15)
b2-a2	0	4	3	2	9 (32,14)
b3-a2 et b2a2	1	0	0	0	1 (3,57)
Total	4 (14,28 %)	9 (32,14%)	9 (32,14%)	6 (21,43%)	28 (100)

## Discussion

Le nombre de patients inclus semble réduit mais nous a néanmoins permis de décrire les différents types de transcrits observés chez les patients LMC suivis au CHU-Campus de Lomé et leurs particularités sur le profil hématologique. Ce nombre peu important s'expliquerait par le fait que l'unité d'hématologie du CHU-Campus ne reçoit probablement pas tous les cas de LMC diagnostiqués au Togo. Il se pose d'une manière générale le problème d'adhésion des patients en Afrique Subsaharienne au suivi médical au long cours [4]. En dix ans, 34cas de LMC avaient été diagnostiqués avec recherche de mutation bcr-abl. Au Sénégal [4] la LMC représentait 16% de l'ensemble des hémopathies chez l'adulte. Les variations de fréquence pourraient s'expliquer par la difficulté de sensibilisation des populations en Afrique noire sur la maladie et surtout l'absence de centralisation des données en matière de cancers en général et d'hémopathies malignes en particulier. L'âge moyen de 42,32±14,87 (extrêmes de 9 et 65 ans) se rapprochait de celui de Mukiibi J.M. et al [5] en Afrique Centrale, Dokekias et al [6] au Congo Brazzaville, de Souza C et al [7] au Brésil qui avaient rapporté respectivement un âge moyen de 38,9 ans; 35 ans et 32 ans. Par contre Tardieu S. et al [8] en France et Sureda et al [9] aux USA avaient trouvé un âge moyen de 50 ans. Ces résultats montrent que la LMC est une affection fréquente chez l'adulte jeune. Même si certains auteurs [10] rapportent une légère prédominance féminine, la prédominance masculine (sex-ratio 1,61 de notre étude) est classiquement observée dans la littérature [5,6,8] et la LMC affecterait indifféremment les deux sexes. Il existait deux types de transcrits de fusion : b3-a2; b2-a2 et trois groupe de patients. Il s'agissait des patients avec transcrit b3-a2 (55,88%), ceux avec transcrits b2-a2 (38,24%) et ceux ayant les deux types (5,88%). Ces taux sont similaires à ceux rapportés par Pignon [2] qui étaient de 55 à 60 % des cas pour b3-a2 ; 25 à 30 % des cas pour b2-a2 et 5 à 10 % des cas pour les patients ayant les deux transcrits. Ces taux s'expliqueraient par la prédisposition des régions a2 ; b2 ; b3 sur les chromosomes 9 et 22 ce qui faciliterait les différents croisements b2-a2 ; b3-a2. Inokuchi et col [11] au Japon sur une population avaient trouvé sur une population de 35 patients, 21 (60%) ayant le transcrit b3-a2, 7(20%) le b2-a2 et 7(20%) les 2 types de transcrit. Le même auteur [12], quelques années plus tôt, sur une population de 57 patients, avait retrouvé que 17 patients avaient le b2-a2, 34 le transcrit b3-a2 et 6 les deux types. Tous ces résultats prouvent que le transcrit le plus fréquent est le b3-a2. Le taux d'Hb moyen de notre série se rapproche de celui de Tolo-Diebkilé et al en Côte d'Ivoire [10] qui était de 8,77g/dl. Il existait également en moyenne une anémie quelle que soit le type de transcrit. Ouédraogo et al [13] au Burkina ont montré une anémie de l'ordre de 89,20 %. Le nombre moyen des globules blancs dans notre étude était de 195.003 ±190,915G/L (extrêmes 6,4 et 971G/L). Dans une série ancienne de 1000 malades à l'hôpital Saint Louis entre 1955 et 1975, Tanzer J. et al [14] ont noté que 30 % de ces malades avaient une leucocytose comprise entre 10 et 100G/L ; 50 % entre 100 et 300G/L et ceux ayant une hyperleucocytose supérieure à 500G/L au moment du diagnostic n'excédaient pas 2 %. Teillet et al en Europe [15] avaient par contre rapporté un taux de 28 % de cas d'hyperleucocytose modérée inférieur à 50G/L au moment du diagnostic. L'hyperleucocytose élevée au diagnostic en Afrique s'expliquerait par le retard de consultation. Le nombre moyen de plaquettes de tous les patients était de 451,664± 204,153G/L (extrêmes 51G et 1116G/L). Tolo-Diebkilé A. et al [10] en Côte d'Ivoire avaient noté un taux de plaquettes qui variait entre 38G/L et 1752G/L avec une moyenne de 360,8G/L. Nous partageons avec Broustet [16] l'idée selon laquelle le nombre de plaquettes est le plus souvent élevé ou normal, ou exceptionnellement diminué. En tenant compte des différents types de transcrit, il existait une relation statiquement significative entre le type de transcrit et la perturbation des paramètres de l'hémogramme. En effet, les anomalies étaient plus profondes chez les patients ayant les deux types de transcrits suivies des patients ayant le b3-a2 et enfin de ceux ayant le b2-a2. Nos résultats se rapprochent de de ceux de Inokuchi et al [12] à propos des plaquettes qui avaient trouvé que le chiffre des plaquettes était significativement plus élevé chez les patients ayant le transcrit b3-a2 que chez ceux ayant le b2-a2 (841,5G/L contre 373,5G/L ; P<0,15). Par contre, ils n'avaient pas de trouvé de relation entre les globules blancs et le taux d'hémoglobine. Le même constant concernant les plaquettes a été fait par Maritat P et al [17]. Nous n'avons pas pu trouver d'explication claire de cette particularité de nos patients pour cette relation significative entre le type de transcrit et les valeurs du taux d'hémoglobine et le nombre des globules blancs.

## Conclusion

Il existe deux types de transcrit au cours de la LMC au Togo. Le b3-a2 et le b2-a2. Il existait une relation statiquement significative entre le type de transcrit le type de transcrit et les anomalies de l'hémogramme. L'hémogramme était plus perturbé chez les patients ayant les deux types de transcrit.

### Etat des connaissances actuelles sur le sujet

- Sur ce sujet, nous savons qu'il existe un transcrit de fusion bcr-abl au cours de la leucémie myéloïde chronique;
- Plusieurs types de transcrits ont été décrits et présents soit individuellement soit simultanément chez les patients;
- La recherche de ce transcrit associé au caryotype est indispensable pour le diagnostic et aussi pour le suivi moléculaire au cours de la leucémie myéloïde chronique.

### Contribution de notre étude à la connaissance

- C'est la première fois qu'une étude est consacrée exclusivement aux différents transcrits observés au cours de LMC au Togo;
- Au Togo comme dans la littérature, deux types de transcrits ont été retrouvés;
- Il existe à l'issue de notre étude une relation statiquement significative entre le type de transcrit et les anomalies de l'hémogramme qui est par ailleurs plus perturbé chez les patients ayant les deux types de transcrit.

## Conflits d’intérêts

Les auteurs ne déclarent aucun conflit d'intérêts.
